# The SDGs and human well-being: a global analysis of synergies, trade-offs, and regional differences

**DOI:** 10.1038/s41598-020-71916-9

**Published:** 2020-09-15

**Authors:** Jan-Emmanuel De Neve, Jeffrey D. Sachs

**Affiliations:** 1 Wellbeing Research Centre, University of Oxford, Oxford, UK; 2 Saïd Business School, University of Oxford, Oxford, UK; 3 Centre for Sustainable Development, Columbia University, New York, USA; 4 Sustainable Development Solutions Network, New York, USA

**Keywords:** Sustainability, Psychology and behaviour

## Abstract

This paper explores the empirical links between achieving the Sustainable Development Goals (SDGs) and subjective well-being. Globally, we find that in terms of well-being, there are increasing marginal returns to sustainable development. Unpacking the SDGs by looking at how each SDG relates to well-being shows, in most cases, a strong positive correlation. However, SDG12 (responsible production and consumption) and SDG13 (climate action) are negatively correlated with well-being. This suggests that in the short run there may be certain trade-offs to sustainable development, and further heterogeneity is revealed through an analysis of how these relationships play out by region. Variance decomposition methods also suggest large differences in how each SDG contributes to explaining the variance in well-being between countries. These and other empirical insights highlight that more complex and contextualized policy efforts are needed in order to achieve sustainable development while optimising for well-being.

## Introduction

This paper explores the empirical links between sustainable development and human well-being. Sustainable development is a broad and easily misunderstood concept^1^, but the term first entered mainstream policy circles with the publishing of the Brundtland report in 1987, in which it was defined as ‘development that meets the needs of the current generation without compromising the ability of future generations to meet their own needs’^2^. Debate continues as to whether sustainable development in practice can live up to its normative promises of economic development, environmental stewardship and social equity^3^. Nonetheless, in 2015 the international community rallied around the idea, and sustainable development gained further exposition with the adoption of the Sustainable Development Goals (SDGs), as part of the broader 2030 Agenda. As the successors to the Millennium Development Goals, the 17 SDGs are a comprehensive set of policy goals that aim to end world poverty and hunger, address climate change and environmental protection, and ensure universal access to healthcare, education and equality^4^.

Parallel discussions have centred around the need to move away from GDP as an assessment of countries’ performance towards measures that better capture levels of happiness and well-being^5^. Subjective well-being measures differ from objective well-being indicators, such as observable health and material outcomes, in that they are based on respondents’ self-evaluations of their own life^6^. Varied research suggests that subjective well-being (SWB) measures, especially life evaluations, reflect underlying well-being^7^. As such, there is now a growing consensus among governments and international institutions that SWB—whilst imperfect^8^—has an important role to play in defining success and, as such, an increasing significance in policy-making^9^.

This research aims to explore the relationship between sustainable development and subjective well-being, with the potential to support future policy-making. To do so, we combine two major data-gathering efforts. We leverage the SDG Index which measures countries’ progress towards achieving the SDGs^10^. We also draw on an item from the Gallup World Poll which is representative of over 98% of the world’s population and asks survey participants to evaluate their lives on a scale of 0–10. The paper begins by discussing the headline positive correlation between the SDG Index and SWB. We analyse the quadratic relationship between the two, demonstrating that a higher SDG Index score correlates more strongly with higher SWB at higher levels of the SDG Index. Globally, we find that there are increasing marginal returns to sustainable development in terms of human well-being. In the next section, the SDG Index is split into its 17 component goals. We analyse the varying relationships with well-being, as well as how these relationships play out by region, finding that two of the environmental goals, Goal 12 (responsible consumption and production) and Goal 13 (climate action), are significantly negatively correlated with SWB. We finish by conducting a variance decomposition analysis to show which goals are most strongly contributing to the variation in well-being between countries and the world’s regions^11^.

Our analysis finds that more complex and contextualised policy efforts are needed in order to simultaneously achieve sustainable development and advance well-being. Human well-being is at the core of the 2030 Agenda^12^: the SDGs aim to ensure that ‘all human beings can fulfil their potential in dignity, equality and in a healthy environment^4^.’ Thus, one might expect to find a positive correlation between the SDGs and SWB. Detailed empirical work, however, shows the relationship to be more nuanced than might first appear. Whilst all SDGs are important, our analysis shows that some are more relevant to well-being than others, and reveals some inherent tensions that involve trade-offs between current and future well-being. Since governments are dependent on the current cohort of electors to decide their fate^13^, more cautious policy is needed to resolve trade-offs, allowing for sustainable development that also optimises for human well-being^14^. Unpacking the SDGs in terms of well-being also shows how their relative importance varies across different regions, highlighting the need for differentiated policy priorities when advancing the 2030 Agenda.

## Data and methods

### Data

The SDG Index (SDGI) was developed in 2015 as a composite system to benchmark the performance of countries across the SDGs. Several indicators are selected to monitor the progress towards each goal, positioning them between the worst (0) and the target outcome (100). The overall SDGI score represents the mean of a country’s total SDG scores, where all goals are weighted equally. The same basket of indicators is used for all countries to generate comparable scores and rankings. For our analysis we use the 2019 SDG index, which includes 114 indicators covering 162 countries^10^. Note that in our analysis, the SDG Index is modified to remove the SWB score, which is one of the indicators for SDG 3 (Health and Well-being). Given the large number of variables that make up the SDG Index, we find that leaving in or taking out the SWB variable does not meaningfully impact any results. Limitations in collecting data for SDG indicators hinder full assessment of progress towards SDGs. There are also issues with the aggregation of goals into a single number^15^, nonetheless there is consensus that the SDGI provides ‘the most comprehensive picture of national progress on the SDGs’^16^.

For our analysis we use life evaluations, the standard measure of well-being used in the World Happiness Report rankings and most other research on the topic^17,18^. We draw on data from the Gallup World Poll, which continually surveys 160 countries representing about 98% of the world’s adult population. The survey item asks respondents to value their current lives on a 0–10 scale, with the worst possible life as 0 and the best possible life as 10. The data is from nationally representative samples, for the years 2016–2018. Some methodological issues remain with subjective well-being measures^6^, but life evaluations are widely recognised as the standard measure of subjective well-being^7,19^. Data on other dimensions of subjective well-being, such as the experience of positive and negative emotions, are analysed separately and can be found in the Supplementary Information section online.

### Methods

The analyses done in this paper rely on standard univariate linear correlations and OLS regressions. In line with the SDGI methodology, where scores are missing for specific goals, we impute using the regional average to avoid losing observations. This is most relevant for goal 14 (Life below water).

The variance decomposition method (dominance analysis) employed in Figs. 4, 5 and 6 is run in Stata using the *domin* command. Dominance analysis calculates the relative contribution to the variance explained in well-being (R-squared) for the 17 SDGs. This is an ensemble method that works by calculating a regression of well-being on every possible combination of the 17 SDGS. The dominance of a goal is calculated as the weighted average marginal contribution to the explained variance that the goal makes across all models in which the goal is included. One important assumption being made in such an analysis is that it forces the SDGs to explain all of the variance in well-being between countries. There are also a number of other important limitations in that the method hinges on there being variance in the first place, and yet the measurements for some SDGs do not vary much.

## Results

### Are the SDGs conducive to human well-being?

**Figure 1:**
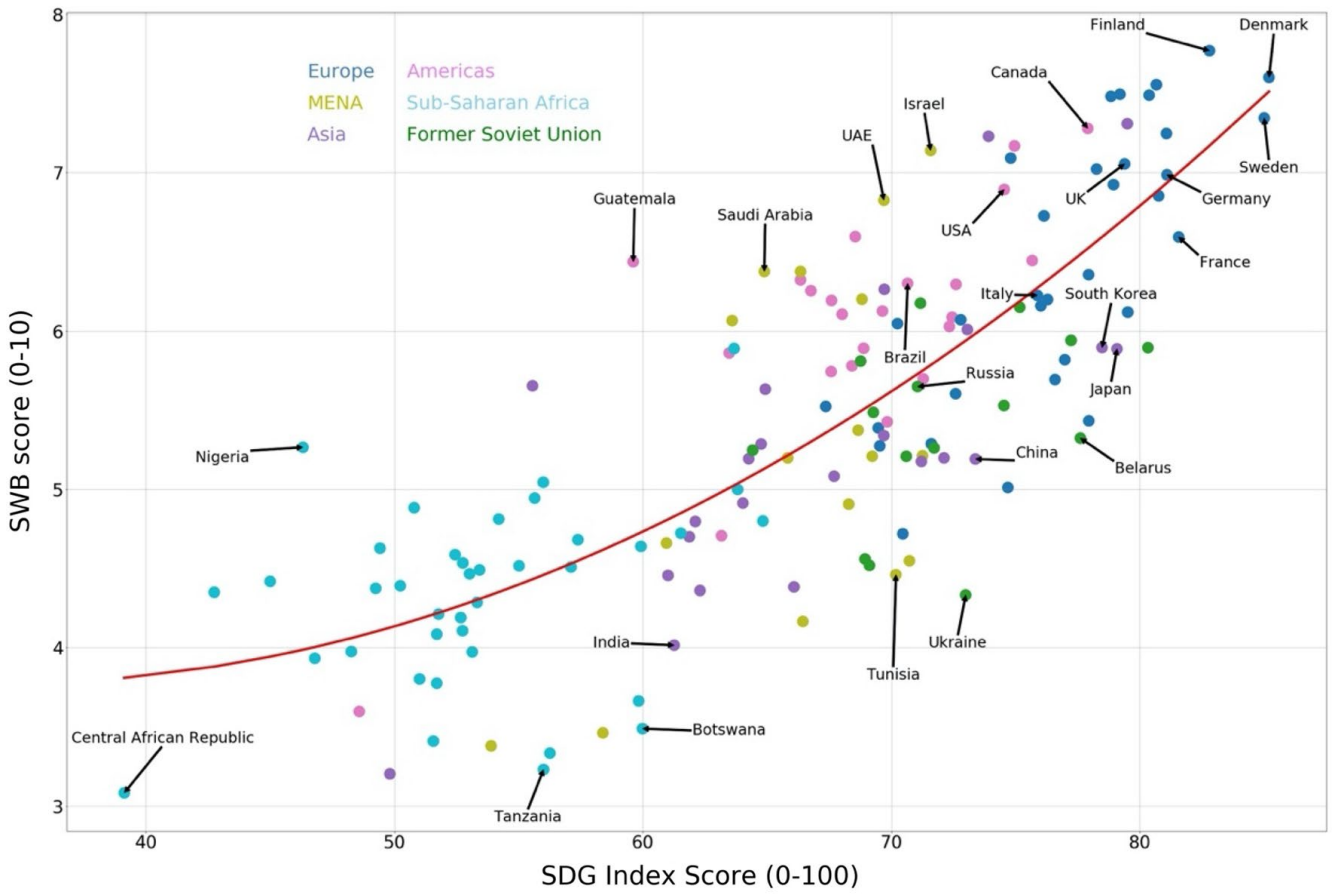
Sustainable development and subjective well-being, a scatterplot for the overall SDGI score (mean of total SDG score, where all goals are weighted equally) and SWB score for all countries in the data set. This scatterplot was produced using matplotlib package (version 3.2.1) in python: https://matplotlib.org.

Figure 1 shows the scatterplot for the SDG Index and SWB for all countries in the dataset. Countries are coded to represent the six regions they belong to: Europe, Middle East and Northern Africa, Americas, Sub-Saharan Africa and Former Soviet Union. The G7 and BRICS countries are labelled as well as some of the outlier countries. The SDG Index and SWB have a highly significant correlation coefficient of 0.79. The countries with a higher SDG Index score tend to do better in terms of subjective well-being (SWB)—with the Nordic countries topping both rankings. Interestingly, the line of best fit is not linear but quadratic indicating that a higher SDG Index score correlates more strongly with higher SWB at higher levels of the SDG Index. Thus, sustainable development results in increasing marginal returns to human well-being.

In the online Supplementary Information section, we show that the quadratic fit is statistically superior compared to a pure linear fit (see Table S1). This is also the case for higher-powered models as borne out when applying the Bayesian information criterion and Akaike information criterion to test the relative quality of model fits (see Table S2). As countries become more developed, a higher SDG Index score is associated with an ever higher SWB score. This suggests that economic activity is more important for well-being at lowers levels of economic development. As countries become richer the well-being of their citizens stagnates unless further economic growth is more sustainable by, for example, addressing inequality and improving environmental quality. The notion of *increasing* marginal returns to sustainable development contrasts starkly with the *decreasing* marginal returns that are typically observed when mapping well-being onto GDP per capita^20^.

Our measure of SWB is an evaluative measure of well-being and the survey responses may differ from emotional measures of well-being, especially when looked at in relation to economic measures such as income and development. As such, in the Supplementary Information section we also report on the relationship between the SDG Index and measures of emotional well-being (see Figure S1 and Figure S2). The Gallup World Poll includes measures of positive emotions such as “enjoyment” and “smile or laugh” as well as negative emotions such as “worry”, “sadness”, “stress”, and “anger”. Correlating an index of positive emotional experiences with the SDG Index scores leads to a correlation coefficient of 0.27—while statistically significant, this indicates a much weaker empirical link between achieving the SDGs and the experience of positive emotions as compared to life evaluations already examined. This is less the case for an index of negative emotional experiences, for which we obtain a correlation coefficient that is − 0.57 suggesting that countries that are not doing well in terms of the SDGs also tend to have populations that are experiencing more negative emotions. In general, these results are in line with the notion that evaluative measures correlate more strongly with economic measures such as income, development, and inequality than emotional measures of well-being^21,22^.

In the Supplementary Information section we list the countries that deviate most from the trend line (see Table S3). The countries significantly above the line of best fit clearly punch above their weight in terms of happiness relative to where the model would expect these countries to be given their scores on the SDG Index, with the reverse being true for those below the line of best fit. These empirical observations indicate that there are a number of aspects that drive human well-being that are not fully captured by the SDGs.

### How does each SDG relate to well-being?

In Table 1 we report on how each SDG correlates with well-being both globally and regionally. As expected from the aforementioned general results, we find that at the global level most SDGs correlate strongly and positively with higher well-being. At the same time, we discover much heterogeneity in how some of the SDGs relate to well-being. In fact, we find SDGs 14 (Life below water), 15 (Life on land), and 17 (Partnerships for the goals) to be generally insignificant. Importantly, we find that SDGs 12 (Responsible consumption and production) and 13 (Climate action) are significantly negatively correlated with human well-being.

**Table 1:** Correlation table for each SDG and subjective well-being (globally and regionally).

		Region						
		All	Europe	Former Soviet Union	Asia	MENA	Sub-Saharan Africa	Americas
**SDG**								
1	No poverty	0.65*	0.49*	− 0.03	0.44	0.22	0.50*	0.76*
2	Zero hunger	0.62*	0.44	0.30	0.41	0.70*	0.23	0.38
3	Good health	0.77*	0.76*	0.40	0.69*	0.82*	0.15	0.89*
4	Quality education	0.64*	0.48*	0.12	0.55*	0.67*	0.14	0.62*
5	Gender equality	0.61*	0.78*	0.55	0.69*	0.75*	− 0.29	0.66*
6	Clean water and sanitation	0.73*	0.69*	0.16	0.83*	0.26	0.00	0.61*
7	Affordable and clean energy	0.69*	0.40	− 0.40	0.71*	0.47	0.51*	0.68*
8	Decent work and economic growth	0.69*	0.62*	0.68*	0.54*	0.77*	0.34	0.61*
9	Industry, innovation and infrastructure	0.80*	0.90*	0.36	0.78*	0.92*	0.35	0.62*
10	Reducing inequality	0.32*	0.71*	0.06	0.12	0.01	0.07	− 0.08
11	Sustainable cities and communities	0.61*	0.74*	0.51	0.56*	0.08	0.00	0.77*
12	Responsible consumption and production	− 0.75*	− 0.69*	− 0.39	− 0.78*	− 0.80*	− 0.26	− 0.51
13	Climate action	− 0.35*	− 0.19	− 0.19	− 0.54*	− − 0.71*	− 0.10	− 0.23
14	Life below water	− 0.02	0.12	0.44	0.18	− 0.14	− 0.02	0.28
15	Life on land	0.03	− 0.06	0.50	− 0.13	− 0.24	− 0.06	0.09
16	Peace, justice and strong institutions	0.69*	0.85*	0.12	0.72*	0.73*	0.06	0.72*
17	Partnerships for the goals	0.16	− 0.03	− 0.28	0.27	0.21	0.04	− 0.02
–	All	0.79*	0.79*	0.37	0.74*	0.55	0.32	0.77*

Univariate correlations where * represents statistical significance at the 1% level. In line with SDG Index methodology, regional averages are used for missing values.

When looking at the relationship between SDGs and well-being by region we detect further levels of heterogeneity in how individual SDGs relate to well-being in different contexts. It is, however, important to note that considering these data by region reduces the number of observations and therefore both the precision of the coefficient and the statistical power to report significant differences. As Fig. 1 revealed visually, there is a stronger link between the SDG Index and well-being at higher levels of economic development. In Table 1 we indeed find that the general correlation between the SDGs and well-being is considerably lower in regions with mostly developing nations. In fact, only for Europe, Asia, and the Americas do we pick up a strong statistically significant correlation between the SDG Index and well-being. When looking at the SDGs individually, we pick up even more variation in how some SDGs are more strongly correlated than others with well-being. Some noteworthy regional results include (1) the important role of SDG 8 (decent work and economic growth) for countries in the former Soviet Union; (2) the relative importance of SDG 9 (industry, innovation and infrastructure) for nations in Europe and the MENA region; and (3) SDG 10 (reducing inequality) is strongly correlated with well-being for the European nations. These regional correlations need to be taken with due caution given the relatively low number of observations available but, taken together, Table 1 paints a vivid picture of the varied and complex ways in which the SDGs relate to human well-being and how these pathways are highly context specific.

### Are there trade-offs between the SDGs and human well-being?

Table 1 reveals that SDG 12 (responsible consumption and production) and SDG 13 (climate action) have, in fact, strong negative correlations with self-reported measures of human well-being. Moreover, these negative correlations appear to hold for each one of the world’s regions and therefore merit more academic and policy attention.

**Figure 2:**
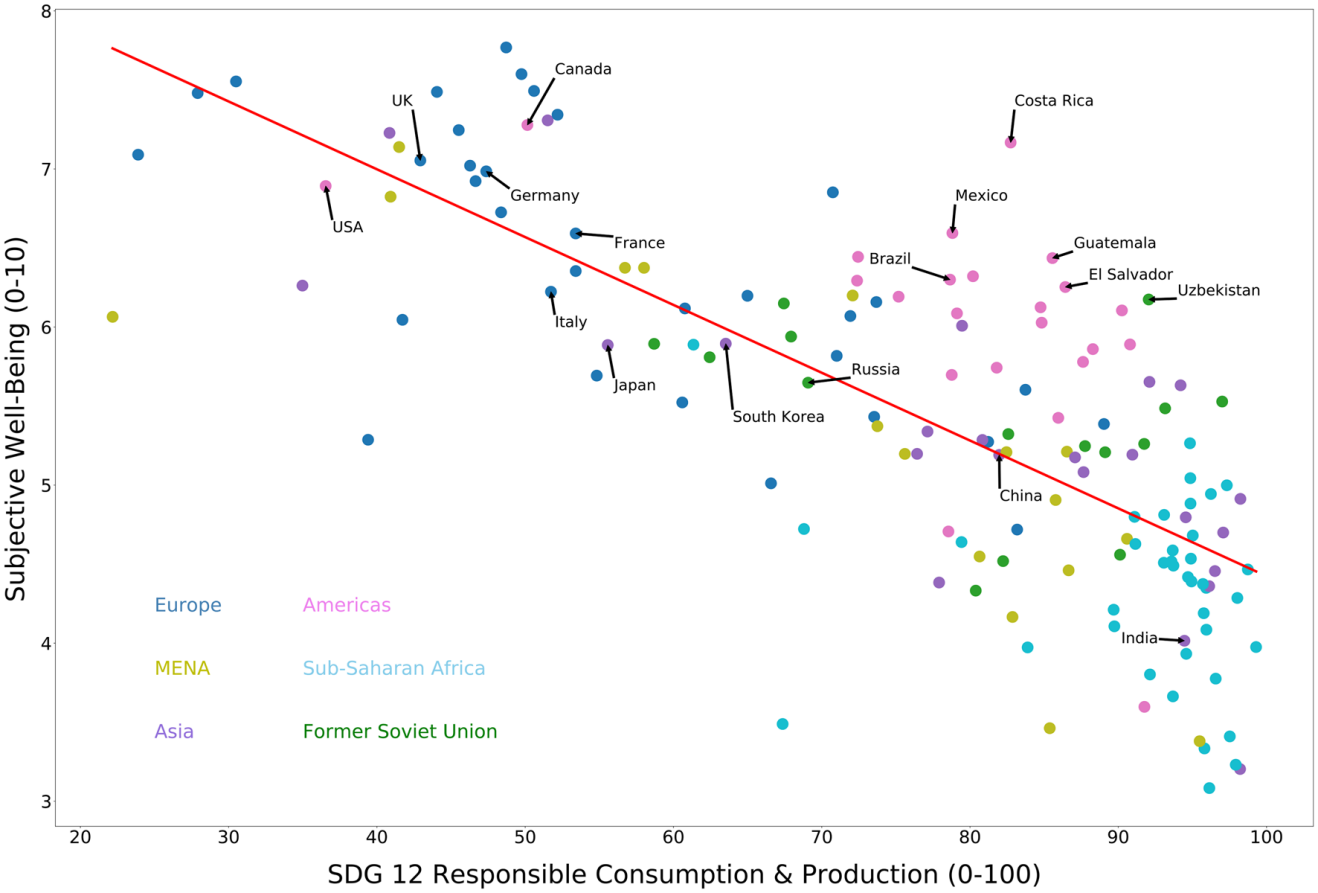
Responsible consumption and production (SDG12) and subjective well-being, a scatterplot for SDG12 score and SWB score for all countries in the data set. This scatterplot was produced using matplotlib package (version 3.2.1) in python: https://matplotlib.org.

SDG12 aims to ensure responsible consumption and production patterns, in order to prevent the over-extraction and degradation of environmental resources. The indicators underlying SDG12 measure the per capita material footprint of each country, accounting for municipal solid waste (kg/year/capita), E-waste generated (kg/capita), production-based SO_2_ emissions (kg/capita), imported SO_2_ emissions (kg/capita), nitrogen production footprint (kg/capita), net imported emissions of reactive nitrogen (kg/capita), non-recycled municipal solid waste (MSW in kg/person/year times recycling rate)^10^. Fig. 2 shows the negative correlation between achieving SDG 12 and subjective well-being. It suggests that countries which have a smaller per capita material footprint—and are therefore performing well on SDG12—are associated with lower levels of SWB. Countries like Canada, meanwhile, have a high material footprint and score badly on SDG12 but perform well in terms of SWB. The relationship between countries’ well-being and material footprint may well be explained by economic development, as countries with higher GDPs tend to produce and consume more, which is usually associated with higher living standards. However, as reported in Table 2, when we control for the general level of economic development, SDG12 continues to correlate negatively with SWB, suggesting that material consumption itself is an important factor explaining this negative correlation. This analysis therefore suggests that advancing on responsible consumption and production may result in a trade-off in terms of average self-reported well-being, at least in the short run. However, it is important to note the handful of countries in the top right-hand corner of Fig. 2 (listed in Supplementary Table S4 online) which run counter to this trend. For example, Costa Rica scores highly in terms of SWB whilst also scoring well on SDG12, suggesting that it is in fact possible to advance human well-being at moderate consumption levels.

**Table 2:** Regression analyses of SDG12 and SDG13 on subjective well-being (controlling for GDP).

	SWB	SWB
SDG 12 (responsible consumption and production)	− 0.522*** (− 4.72)	
SDG 13 (climate action)		0.108 (1.54)
GDP per capita	0.264** (2.39)	0.783*** (11.12)
Adjusted R^2^	0.577	0.520
N	147	147

T-statistics are in parentheses. * represents significance at 10% level. ** represents significance at 5% level. *** represents significance at 1% level.

**Figure 3:**
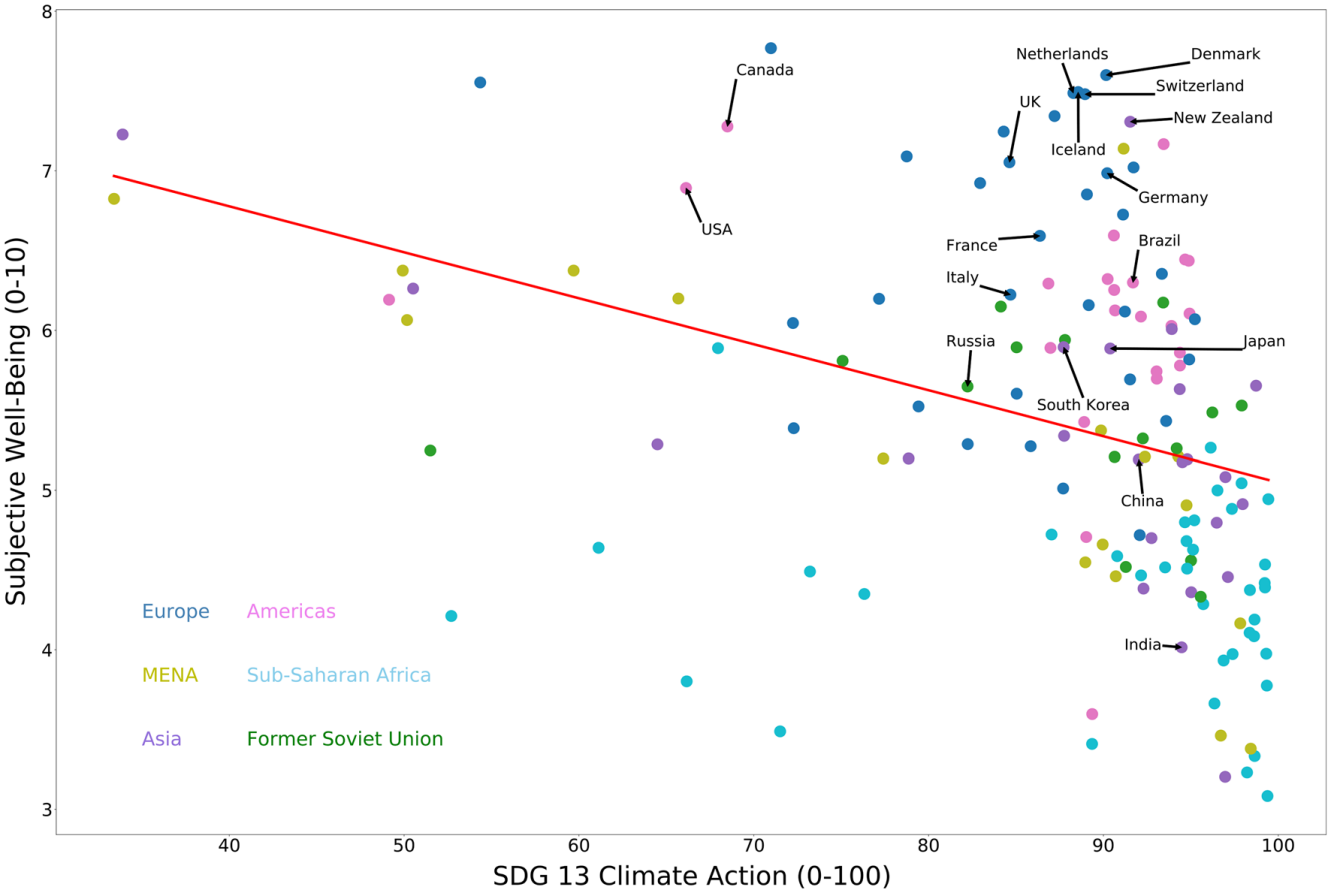
Climate action (SDG13) and subjective well-being, a scatterplot for SDG13 score and SWB score for all countries in the data set. This scatterplot was produced using matplotlib package (version 3.2.1) in python: https://matplotlib.org.

SDG 13 asks that countries take urgent action to combat climate change and its impacts by curbing emissions. It measures countries’ energy-related CO_2_ emissions per capita (tCO_2_/capita), imported CO_2_ emissions, technology adjusted (tCO_2_/capita), people affected by climate-related disasters (per 100,000 population), CO_2_ emissions embodied in fossil fuel exports (kg/capita), effective carbon rate from all non-road energy, excluding emissions from biomass (€/tCO_2_)^10^. In general, countries that have lower emissions—and are therefore performing well on SDG13—tend to have lower levels of subjective well-being. As was the case with SDG 12, countries that are more economically developed tend to pollute more while also having higher well-being. In contrast with SDG12, however, we find that accounting for the general level of economic development turns a negative correlation into an insignificant one as reported in Table 2. This suggests that the underlying measures for climate action are strongly correlated with the level of economic development in the first place which, in turn, drives the relationship with well-being. Again, there are a handful of countries in the top right of Fig. 3 (listed in Supplementary Table S5 online), which appear to be resolving the trade-off, performing well on SDG13 whilst maintaining high levels of SWB.

### Variance decomposition analysis of the SDGs in relation to well-being

In this section, we apply variance decomposition to explore the relative importance of each SDG in explaining the variance in well-being between countries. This method, called “dominance analysis”, investigates the relative contribution to the variance explained in well-being (R^2^) for a given set of predictors—in this case the 17 SDGs^11^.

**Figure 4:**
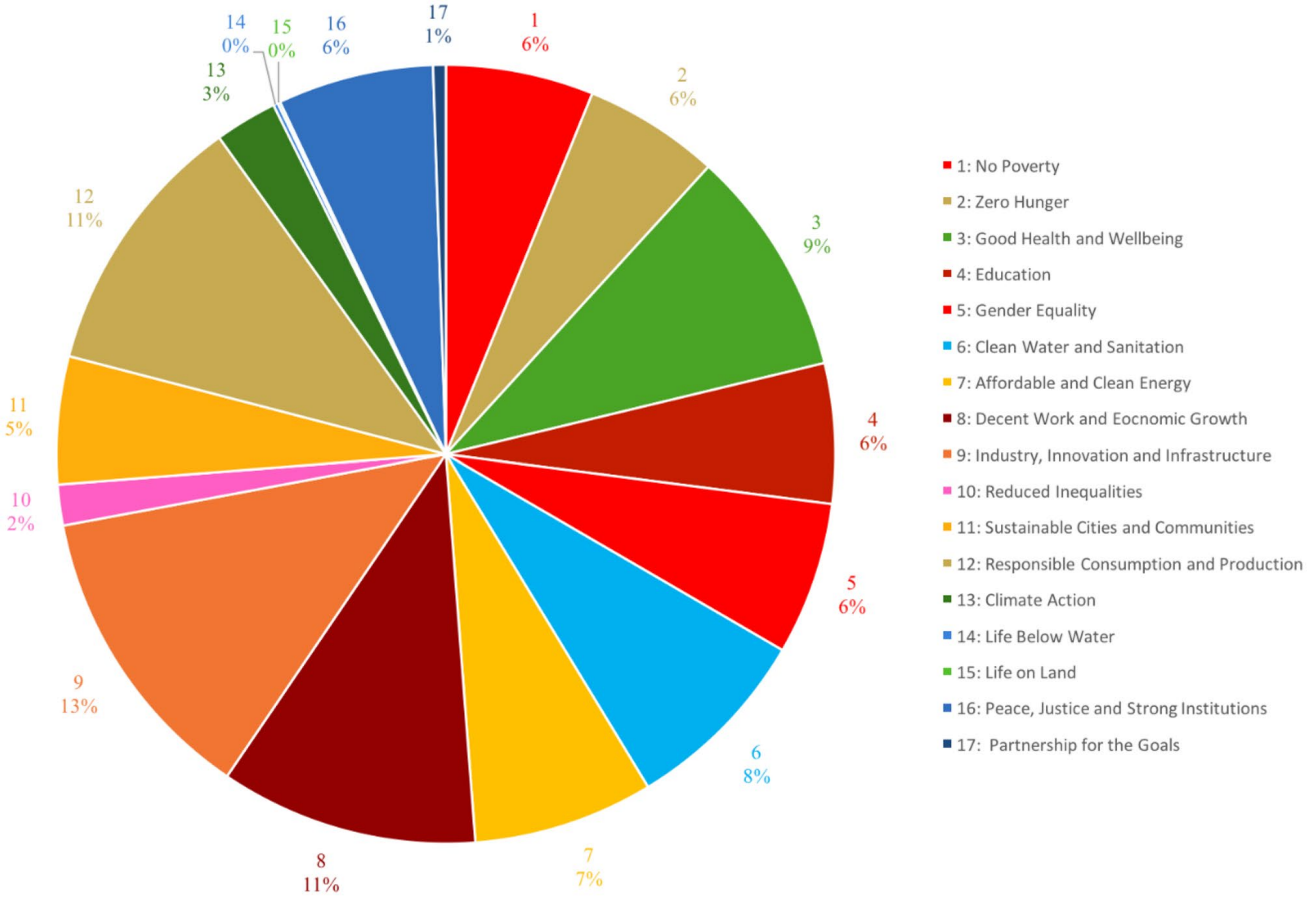
Relative importance of SDGs in explaining the variance in subjective well-being between countries.

Figure 4 presents the results of the variance decomposition and suggests large differences in how each SDG contributes to explaining the variance in well-being between countries. This figure paints a picture that aligns closely with the correlation coefficients reported in Table 1. SDGs 10, 14, 15 and 17 would appear to contribute negligibly to explaining variation in well-being across the globe. On the other hand, the greatest explanatory power seems to lie with SDGs 3, 8, 9, and 12. SDG 8 (decent work and economic growth), SDG 9 (industry, innovation and infrastructure), and SDG 12 (responsible consumption and production) each explain 10% or more of the variance. It is important to note, of course, that SDG 12 (as well as SDG 13) are negatively correlated with well-being, as was shown earlier on in Table 1.

### Variance decomposition analysis of regional SDG groups in relation to well-being

**Figure 5:**
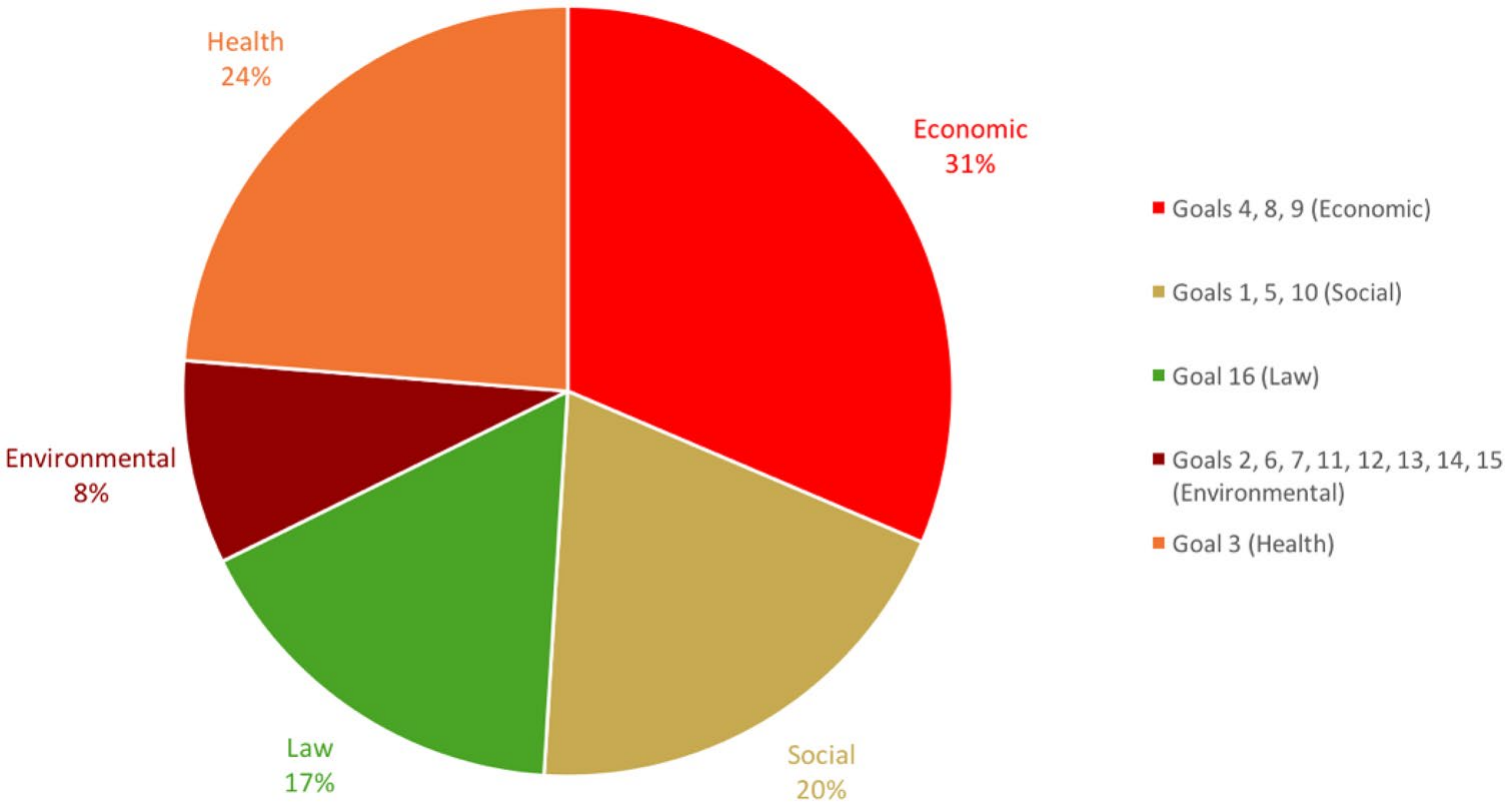
Relative importance of SDG groups in explaining the variance in subjective well-being between countries.

**Figure 6:**
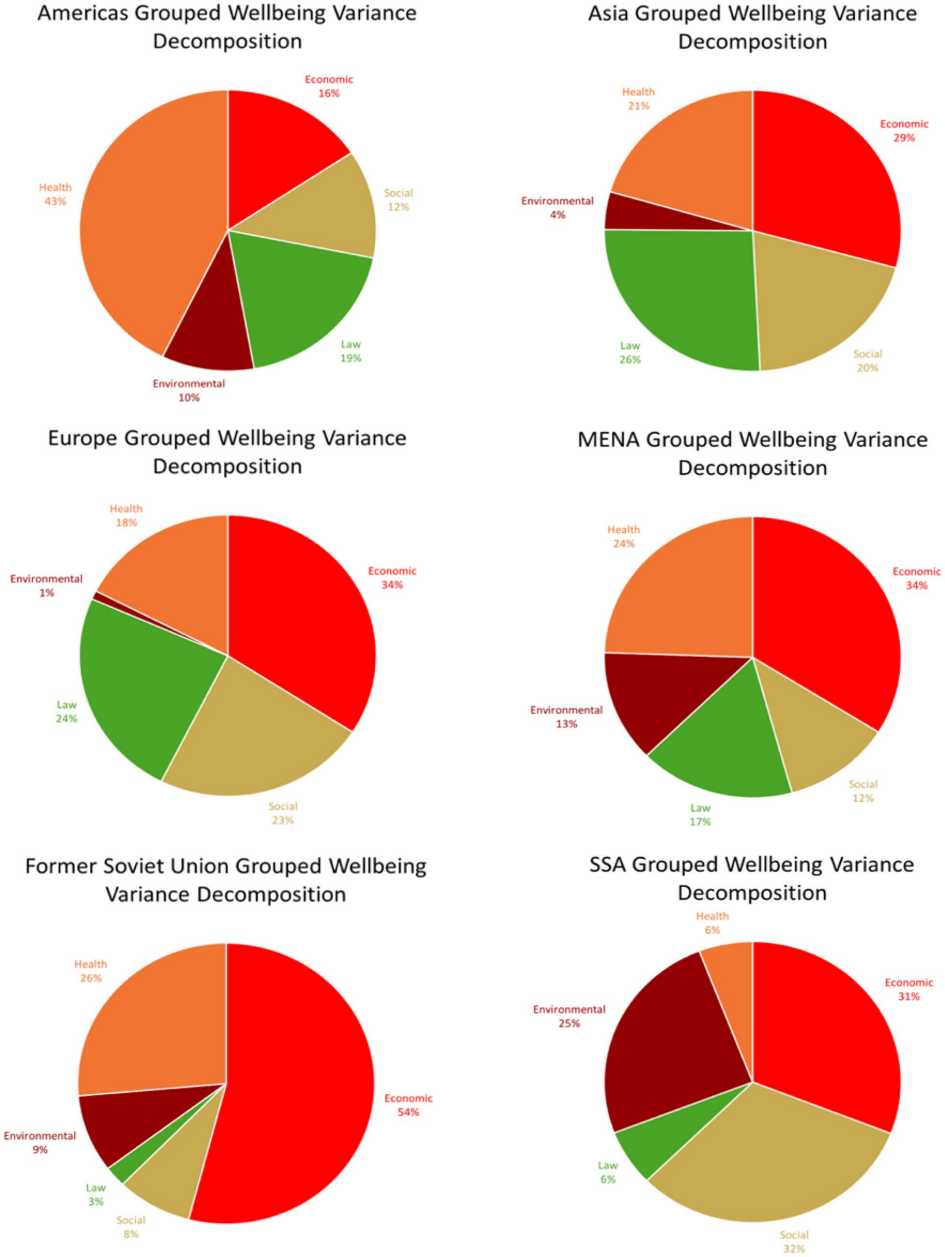
Relative importance of SDG groups in explaining regional subjective well-being variance.

In these analyses, we group the SDGs into Economic (4, 8, 9), Social (1, 5, 10), Health (3), Law (16), and Environmental goals (2, 6, 7, 11, 12, 13, 14, 15). Figure 5 first shows the results for how well these SDG groups explain the variance between all countries. In Fig. 6 we show the results by region. The general takeaway from the regional variance decomposition analyses is that there is much regional heterogeneity hidden behind a global analysis, with the regional context driving which SDGs are most important in explaining the variance in well-being between countries in the region. In Europe (N = 33), and especially in the countries of the former Soviet Union (N = 15), we find the great importance of the Economic SDGs in explaining regional variation in well-being. In Asia (N = 23) we find a fairly balanced role for the Economic, Law, Social, and Health SDG groups in explaining regional differences in well-being. In the Americas (N = 23) we find that Health plays the most important role in driving regional variation in well-being. The results for Sub-Saharan Africa (N = 38) point towards the Social SDGs playing the key role in explaining regional differences. For the countries in the MENA region (N = 17) we find a more balanced picture with the Health and Economic SDGs driving most of the variation, but an important role as well for the Social, Law, and Environmental SDGs.

## Discussion

This paper has studied the empirical relationship between the Sustainable Development Goals and subjective well-being using data from the SDG Index and the Gallup World Poll. We find a strong correlation between achieving sustainable development and self-reported measures of well-being. Moreover, our analyses indicate that there are increasing marginal returns to sustainable development in terms of well-being.

While most SDGs are positively correlated with well-being, our analysis reveals that SDG12 (responsible consumption and production) and SDG13 (climate action) are negatively correlated with SWB^23^. These findings are perhaps unsurprising: the world economy has long relied on economic growth and the consumption of natural resources to generate human welfare at the expense of environmental sustainability^3,24^. Today, however, it is increasingly clear that if we are to avoid ecological collapse, we must bring our consumption of natural and material resources within ecological limits^25,26^. This transformation is captured by SDG12 and SDG13; it will involve real reductions in emissions, and quantitative as well as qualitative changes to consumption and production patterns^27^. In particular, high income countries must reduce their ecological footprint to allow for increased consumption in economically developing countries, where it is necessary for meeting basic needs^23,28^. This is not an easy task given that our growth-driven economic system is reliant on ever-increasing consumption and production to provide employment and support livelihoods^29^. Thus, under current structures, advancing on SDG12 and SDG13 could have serious socio-economic consequences and, as such, negatively impact well-being levels, particularly those of the most vulnerable^27^. Given that lowering well-being erodes support for incumbent governments, this makes such policies even more difficult to implement^13^. More cautious policies are therefore needed to ensure that progress towards SDG12 and SDG13 also safeguards livelihoods and well-being^30,31^.

Nevertheless, environmental stewardship does not necessarily entail reductions in well-being. Varied research has shown the importance of environmental integrity for human well-being: for instance, subjective well-being is negatively influenced by poor air quality^32^; people are willing to pay for observably cleaner air^33^; and there is evidence to suggest that being exposed to nature improves mental health^34^. Furthermore, as we have shown elsewhere there is a strong positive correlation between SWB and the Environmental Protection Indicator (a measure which is much wider in scope than the environmentally-oriented SDGs, covering a broad range of issues such as biodiversity and eco-systems, climate and energy, air pollution, water resources, agriculture, heavy metals, water and sanitation, and air quality)^35^. These research insights indicate that well-being is correlated with the long-term outcomes of environmental policies, even if it is not necessarily positively correlated with the short-run efforts required of such policies.

The challenge for policy-makers is thus to resolve the short-term trade-off by de-coupling human well-being improvements from the consumption of natural resources and GHG emissions^36^. A recent report by the OECD attempts to address this challenge by proposing climate change mitigation through a well-being lens, putting people at the centre of climate action^37^. The outlier countries highlighted in our analysis (see Supplementary Table S4 and S5 online) that are performing well on SDG12 and SDG13, whilst also achieving high levels of well-being, indicate that there might be pathways to improving well-being that do not hinder environmental sustainability^38,39^. These countries represent a proportional mix of relatively large and small countries across the world. For example, Germany has invested heavily in renewable energy infrastructure^40^, providing ‘green jobs’ while simultaneously reducing emissions. The combination of carbon taxes and incentives for renewable energy, combined with ambitious social policy, has allowed the Nordic countries to transition away from fossil fuels, without punishing low-income families with higher energy bills^41,42^. Equally, Costa Rica is among the top countries for investment in new renewable power and fuels relative to GDP, and has committed to achieving carbon neutrality starting from 2021^43^. It thus offers an alternative model for developing countries to avoid the Western carbon-intensive development path^44^. Interestingly, many Latin American countries with warmer climates and a lower propensity to engage in international trade^36^ perform strongly in terms of self-reported well-being whilst also scoring highly in terms of SDG12 (sustainable consumption and production), supporting the notion that human well-being decouples from environmental impact beyond minimum levels of consumption^39^. More research is needed to better understand the development trajectories of these countries and the policy mechanisms which allow for synergies between well-being and ecological sustainability^36^. Policies such as investment in public services to moderate private consumption^27^ and harnessing productivity gains to reduce working hours^45^ have been proposed. There is also increasing evidence from sustainable cities that supports the notion that it is possible to mitigate environmental issues and simultaneously improve quality of life^46^.

Trade-offs between the SDGs and SWB can also arise as a result of interactions between different SDGs. In particular, SDGs 11, 13, 14, 16, and 17 continue to have negative trade-offs and non-associations with other SDGs^47^. The highly positive links we identified between goals 11 and 16 and human well-being may possibly compensate for these intra-SDG trade-offs, but policy-makers may find pursuing SDGs 13, 14, and 17 more difficult due to the negative or insignificant correlation with the well-being of current generations. Needless to say, however, that the urgency of climate change does require action to ensure the well-being of future generations^48,49^.

Regional analyses have revealed that what accounts for human well-being varies greatly according to regional and socio-economic context; policy efforts must therefore be differentiated. For example, we find that while in Europe reducing inequalities significantly contributes to well-being, poverty reduction is more important in sub-Saharan Africa. These findings complement a recent study of SDG interactions, which finds poverty alleviation in low-income countries and reducing inequalities in high-income countries to have compounded positive effects on all SDGs^50^, thus helping to support the prioritization of these SDGs according to region. Our findings confirm that general analyses often hide important heterogeneity; moreover, we recognise that the picture becomes even more nuanced at the local level, which is increasingly the site where sustainable development policy is implemented^51^. Importing policy models or ‘best practices’ from elsewhere without a deep understanding of the local context can often obscure effective policy-making on sustainable development issues^52^. As explored in the policy mobilities literature, there is often a mismatch between local governance structures and top-down frameworks like the SDGs which can hinder the overall success of such agendas^53^. Where policies are too insensitive to specific local variations, the goals of sustainable development can be squandered. Therefore, a more comprehensive understanding of how the SDGs can be implemented at the local level is critical^54^ in order to advance the 2030 agenda such that both people and planet can thrive.

Our analysis is of course limited by data gaps for several SDG indicators, we therefore emphasize the need for increased transparency and co-operation from governments. Regional analyses are limited by the relatively low number of observations available. It is also important to reiterate that variance decomposition analyses are constrained by their methods and the number of observations. As such, these results are meant to be seen as cautious exploration of large-scale trends that are correlational in nature and thus open to potential reverse causality and omitted variable bias. Our aim here is to stimulate thinking and further research on how the SDGs relate to human well-being—and to show that general analyses may hide important heterogeneity when looking at individual SDGs and in the context of different regions. We recognize that in addition to the macro-level statistical analysis conducted here, more research and careful qualitative analysis is needed to understand local complexities and how they interact with the SDG framework.

We have studied the link between the SDGs and SWB of current generations. Future research should investigate the extent to which self-reported SWB metrics account for the well-being of future generations. This is especially relevant when considering SDG 12 (responsible consumption and production) and SDG 13 (climate policy). Implementing these policies requires intergenerational reciprocity, the idea that we must act on the behalf of future generations, which has in turn been shown to depend on the behavior of previous generations^55^. This work also does not address international dynamics. The sustainable development of a country may come at a cost to other countries, or the actions of countries may influence the well-being in others^56^. Furthermore, the model of linking SDGs with well-being assumes only direct relationships, whereas recent work shows that addressing SDGs have knock-on effects for other SDGs^57^.

A potential dynamic that is worthwhile highlighting is the extent to which the well-being of populations may itself exert influence on their country’s approach to development. Changes in well-being have been documented to have wide-ranging effects on economic, social, and health outcomes^58^. Given these objective benefits of subjective well-being there is an urgent need to combine the SDG and SWB research and policy agendas to generate solutions that advance human well-being, without compromising the environmental integrity of our planet.

## Supplementary information

Supplementary Information.

## Data Availability

Data from the SDG index is freely available and can be downloaded from www.sdgindex.org. The Gallup World Poll data is not freely available however the data used in this analysis is made available in the online appendix for the World Happiness Report from https://worldhappiness.report.

## References

[1] Hopwood B, Mellor M, O’Brien G (2005). Sustainable development: mapping different approaches. Sustain. Dev..

[2] World Commission on Environment and Development (1987). Our Common Future.

[3] Krueger R, Gibbs D (2007). The Sustainable Development Paradox: Urban Political Economy in the United States and Europe.

[4] United Nations. *Transforming Our World: The 2030 Agenda for Sustainable Development *(2015).

[5] Stiglitz, J. E., Sen, A. & Fitoussi, J.-P. *Report by the Commission on the Measurement of Economic Performance and Social Progress* (2009).

[6] OECD (2018). For Good Measure: Advancing Research on Well-being Metrics Beyond GDP.

[7] Frijters P, Clark AE, Krekel C, Layard R (2020). A happy choice: wellbeing as the goal of government. Behav. Public Policy.

[8] Benjamin DJ, Cooper KB, Heffetz O, Kimball MS (2019). Self-reported wellbeing indicators are a valuable complement to traditional economic indicators but aren’t yet ready to compete with them. Behav. Public Policy.

[9] O'Donnell, G., Deaton, A., Halpern D., Durand, M. & Layard. R. *Wellbeing and Policy Report* (Legatum Institute, 2014).

[10] Sachs, J., Schmidt-Traub, G., Kroll, C., Lafortune, G. & Fuller, G. *Sustainable Development Report 2019* (Bertelsmann Stiftung and Sustainable Development Solutions Network, 2019).

[11] Azen R, Budescu DV (2003). The dominance analysis approach for comparing predictors in multiple regression. Psychol. Methods.

[12] Patel V (2018). The Lancet Commission on global mental health and sustainable development. Lancet.

[13] Ward G (2019). Happiness and voting: evidence from four decades of elections in Europe. Am. J. Polit. Sci..

[14] Bennett NJ (2019). Towards a sustainable and equitable blue economy. Nat. Sustain..

[15] Diaz-Sarachaga JM, Jato-Espino D, Castro-Fresno D (2018). Is the Sustainable Development Goals (SDG) index an adequate framework to measure the progress of the 2030 agenda?. Sustain. Dev..

[16] Tracking progress on the SDGs. *Nat. Sustain.* **1,** 377. 10.1038/s41893-018-0131-z (2018)

[17] Helliwell, J., Layard, R., Sachs, J. & De Neve, J.-E. *World Happiness Report 2020* (Sustainable Development Solutions Network, 2020).

[18] Helliwell, J. Measuring and using happiness to support public policies. *NBER Working Paper 26529*. https://www.nber.org/papers/w26529 (2019).

[19] Oswald AJ, Wu S (2010). Objective confirmation of subjective measures of human well-being: evidence from the U.S.A.. Science.

[20] Easterlin RA, McVey LA, Switek M, Sawanfa O, Zweig JS (2010). The happiness-income paradox revisited. Proc. Natl. Acad. Sci..

[21] Kahneman D, Deaton A (2010). High income improves evaluation of life but not emotional well-being. Proc. Natl. Acad. Sci..

[22] Powdthavee N, Burkhauser RV, De Neve J-E (2017). Top incomes and human well-being: evidence from the Gallup World Poll. J. Econ. Psychol..

[23] O’Neill DW, Fanning AL, Lamb WF, Steinberger JK (2018). A good life for all within planetary boundaries. Nat. Sustain..

[24] Dietz T, Rosa EA, York R (2009). Environmentally efficient well-being: rethinking sustainability as the relationship between human well-being and environmental impacts. Hum. Ecol. Rev..

[25] IPCC. *Global warming of 1.5 °C. An IPCC Special Report on the Impacts of Global Warming of 1.5 °C Above Pre-Industrial Levels and Related Global Greenhouse Gas Emission Pathways, in the Context of Strengthening the Global Response to the Threat of Climate Change, Sustainable Development, and Efforts to Eradicate Poverty* (2018).

[26] Meadows DH, Randers J, Meadows DL (2004). The Limits to Growth: the 30-Year Update.

[27] Bengtsson M, Alfredsson E, Cohen M, Lorek S, Schroeder P (2018). Transforming systems of consumption and production for achieving the sustainable development goals: moving beyond efficiency. Sustain. Sci..

[28] Scherer L (2018). Trade-offs between social and environmental sustainable development goals. Environ. Sci. Policy.

[29] Jackson T (2009). Prosperity Without Growth: Economics for a Finite Planet.

[30] McCauley D, Heffron R (2018). Just transition: integrating climate, energy and environmental justice. Energy Policy.

[31] Alves MWFM, Mariano EB (2018). Climate justice and human development: a systematic literature review. J. Clean. Prod..

[32] Levinson A (2012). Valuing public goods using happiness data: the case of air quality. J. Public Econ..

[33] Luechinger S (2009). Valuing air quality using the life satisfaction approach. Econ. J..

[34] Guite HF, Clark C, Ackrill G (2006). The impact of the physical and urban environment on mental well-being. Public Health.

[35] De Neve J-E, Sachs J, Helliwell J, Layard R, Sachs J, De Neve J-E (2020). Sustainable development and human well-being. World Happiness Report 2020.

[36] Roberts JT (2020). Four agendas for research and policy on emissions mitigation and well-being. Glob. Sustain..

[37] OECD (2019). Accelerating Climate Action: Refocusing Policies Through a Well-being Lens.

[38] Knight KW, Rosa EA (2011). The environmental efficiency of well-being: a cross-national analysis. Soc. Sci. Res..

[39] Steinberger JK, Roberts JT (2010). From constraint to sufficiency: the decoupling of energy and carbon from human needs, 1975–2005. Ecol. Econ..

[40] Lehr U, Nitsch J, Kratzat M, Lutz C, Edler D (2008). Renewable energy and employment in Germany. Energy Policy.

[41] Nordic Council of Ministers. *Nordic Climate Policy—A Case Study on Efficient Policy Measures*. 10.6027/NA2014-906 (2014)

[42] Martela F, Greve B, Rothstein B, Saari J, Helliwell J, Layard R, Sachs J, De Neve J-E (2020). The Nordic exceptionalism: what explains why the Nordic Countries are constantly among the happiest in the world. World Happiness Report 2020.

[43] OECD (2015). Climate Change Mitigation: Policies and Progress.

[44] Lamb WF (2016). Which countries avoid carbon-intensive development?. J. Clean. Prod..

[45] Knight KW, Rosa EA, Schor JB (2013). Could working less reduce pressures on the environment? A cross-national panel analysis of OECD countries, 1970–2007. Glob. Environ. Change.

[46] Bulkeley H (2013). Cities and Climate Change.

[47] Kroll C, Warchold A, Pradhan P (2019). Sustainable development goals (SDGs): are we successful in turning trade-offs into synergies?. Palgrave Commun..

[48] Stern NH (2015). Why Are We Waiting? The Logic, Urgency, and Promise of Tackling Climate Change.

[49] Stern N (2018). Public economics as if time matters: climate change and the dynamics of policy. J. Public Econ..

[50] Lusseau D, Mancini F (2019). Income-based variation in sustainable development goal interaction networks. Nat. Sustain..

[51] Carr C (2015). Raising sustainability/Mobilising sustainability: why European sustainable urban development initiatives are slow to materialise/Territorial cohesion as a vehicle of sustainability/Sustainable urban development and the challenge of global air transport nodes and spatial integration/Distorted density: where developers and non-governmental organizations on sustainable urban development agree/Overcoming politics with markets? The co-production of sustainable development in urban and regional planning. Plan. Theory Pract..

[52] Temenos C, McCann E (2012). The local politics of policy mobility: learning, persuasion, and the production of a municipal sustainability fix. Environ. Plan. Econ. Space.

[53] Carr C (2014). Discourse yes, implementation maybe: an immobility and paralysis of sustainable development policy. Eur. Plan. Stud..

[54] McLean BL, Borén T (2015). Barriers to implementing sustainability locally: a case study of policy immobilities. Local Environ..

[55] Wade-Benzoni KA (2002). A golden rule over time: reciprocity in intergenerational allocation decisions. Acad. Manag. J..

[56] Schmidt-Traub G, Moff H, Bernlöhr M (2019). International Spillovers and the Sustainable Development Goals.

[57] Pradhan P, Costa L, Rybski D, Lucht W, Kropp JP (2017). A systematic study of sustainable development goal (SDG) interactions. Earths Future.

[58] De Neve J-E, Diener E, Tay L, Xuereb C, Helliwell J, Layard R, Sachs J (2013). The objective benefits of subjective well-being. World Happiness Report 2013.

