# Supplementary material for: The SDGs and human well-being: a global analysis of synergies, trade-offs, and regional differences
Source: Sci Rep. 2020 Sep 15;10:15113. doi: 10.1038/s41598-020-71916-9 (PMC7492223; doi:10.1038/s41598-020-71916-9)

Supplementary Information for

The SDGs and human well-being: a global analysis of synergies, trade-offs, and regional differences

Jan-Emmanuel De Neve*[1,2] and Jeffrey D. Sachs[3,4]

[1]Wellbeing Research Centre, University of Oxford
[2]Saïd Business School, University of Oxford
[3]Centre for Sustainable Development, Columbia University
[4]Sustainable Development Solutions Network
*Correspondence to jan-emmanuel.deneve@sbs.ox.ac.uk

Supplementary Table S1: Model curvature test for SDG Index on SWB

	**SWB**	**SWB**
**SDG Index**	0.7865***	-0.8926
	(15.44)	(-1.53)
**(SDG Index)^2**	-	1.6852***
		(2.90)
**Adjusted R^2**	0.616	0.634
**N**	149	149

Note: *** means significant at the 1% level and t-statistics are given in parentheses

Supplementary Table S2: Model fit by power

**Model fit by power**	**Akaike information criterion (AIC) score**	**Bayesian information criterion (BIC) score**
**Linear**	313.4523	319.4602
**quadratic**	307.1310	316.1428
**3^d power**	309.1064	321.1221
**4th power**	311.0866	326.1063

Supplementary Table S3: Country outliers relative to model line of best fit

Country	Distance above fit line	Country	Distance below fit line
Guatemala	1.73	Ukraine	1.61
Israel	1.36	Botswana	1.24
Nigeria	1.28	Tanzania	1.23
Saudi Arabia	1.25	Tunisia	1.18
UAE	1.24	Belarus	1.16
Pakistan	1.22	Syria	1.16
Australia	1.19	Iran	1.15
Mexico	1.12	Rwanda	1.14
Qatar	1.11	Bulgaria	1.12
Panama	1.06	Egypt	1.10

Supplementary Table S4: Top 10 countries closest to the top right for Figure 2 (by Euclidean distance).

Country	Euclidean Distance from (10,100)*
Costa Rica	33
Guatemala	38
Uzbekistan	39
El Salvador	40
Mexico	40
Nicaragua	40
Colombia	42
Panama	42
Jamaica	42
Ecuador	43

Note: The well-being scores are multiplied by 10 so they are on same scale as the SDGs

Supplementary Table S5: Top 10 countries closest to the top right for Figure 3 (by Euclidean distance).

Country	Euclidean Distance from (10,100)*
Denmark	26
Switzerland	28
Iceland	28
Netherlands	28
New Zealand	28
Costa Rica	29
Sweden	29
Israel	30
Ireland	31
Germany	32

Note: The well-being scores are multiplied by 10 so they are on same scale as the SDGs

Supplementary Figure S1: Sustainable development and positive affect

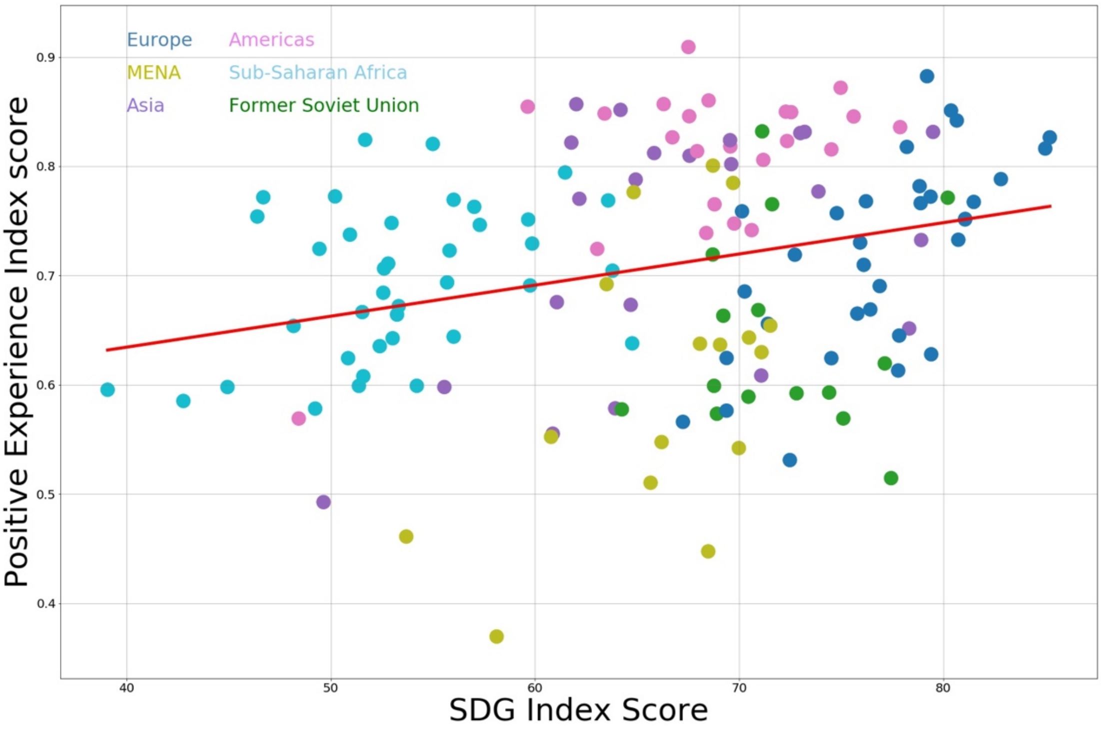

Supplementary Figure S2: Sustainable development and negative affect

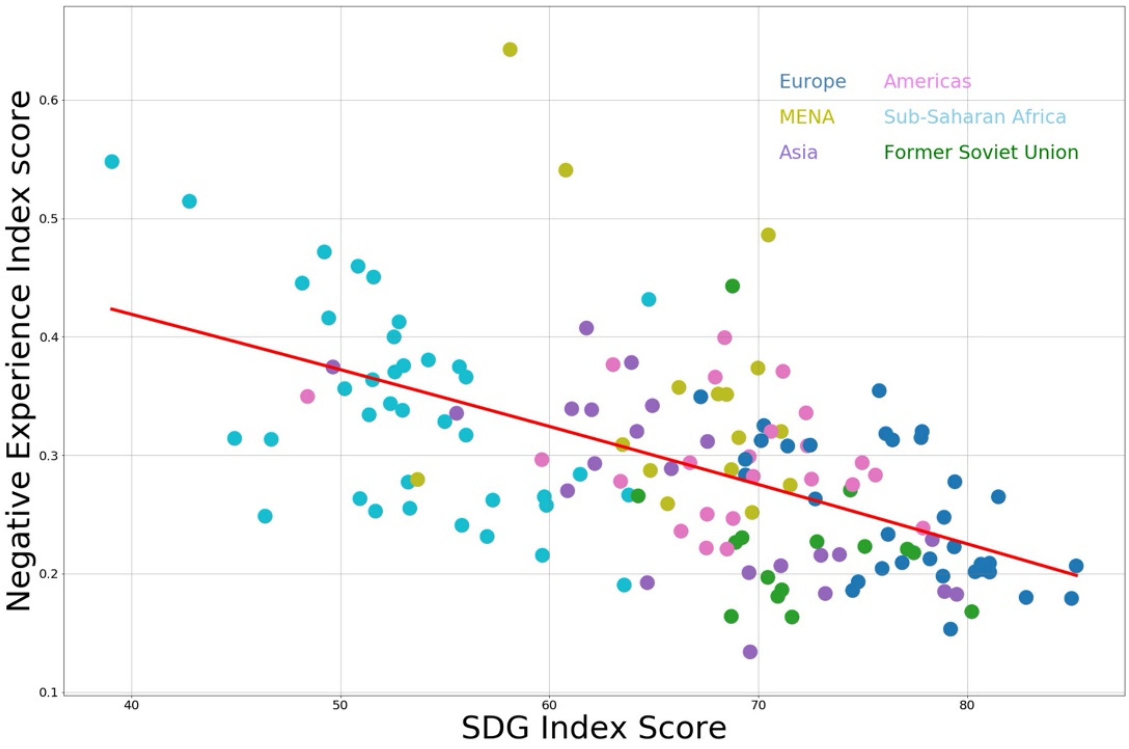

Supplement: Supplementary file 1 — Supplementary Information. [file 41598_2020_71916_MOESM1_ESM.pdf]
